# Reassessing Hybridisation in Australian *Tetragonula* Stingless Bees Using Multiple Genetic Markers

**DOI:** 10.1002/ece3.70912

**Published:** 2025-01-29

**Authors:** James P. Hereward, Tobias J. Smith, Ros Gloag, Dean R. Brookes, Gimme H. Walter

**Affiliations:** ^1^ School of the Environment The University of Queensland Brisbane Queensland Australia; ^2^ School of Life and Environmental Sciences The University of Sydney Sydney New South Wales Australia; ^3^ USDA ARS, Australian Biological Control Laboratory (ABCL), CSIRO, Ecosciences Precinct Dutton Park Australia

**Keywords:** gene flow, genetic marker, hybridisation, Meliponini, species, stingless bee

## Abstract

We re‐examined reports of hybridisation in three cryptic stingless bee species in the genus *Tetragonula* in South East Queensland, Australia ( *T. carbonaria* , *T. davenporti* and *T. hockingsi* ). Previous studies on this group using microsatellite markers proposed that hybridisation occasionally takes place. In contrast, we find that using 1745 SNPs we could reliably separate the three species, with no evidence of contemporary (or recent) hybridisation. We found identical amplicon sequences of the nuclear gene *EF1alpha* across most individuals of the three species, but low and moderate species‐specific polymorphisms in the nuclear gene *Opsin* and the mitochondrial *16S* rRNA gene, respectively, with no cases of mito‐nuclear discordance at these genes. We confirm that nuclear divergence across these species is low, based on 10–26 kb of non‐coding sequence flanking *EF1alpha* and *Opsin* (0.7%–1% pairwise difference between species). However, we find mitogenomes to be far more diverged than nuclear genomes (21.6%–23.6% pairwise difference between species). Based on these comprehensive analyses of multiple marker types, we conclude there is no ongoing gene flow among the *Tetragonula* species of South East Queensland, despite their morphological similarity to one another and the low nuclear divergence among them. The higher resolution provided by multiple SNP markers may lead to lower estimates of contemporary hybridisation more generally.

## Graphical abstract

We reassessed previous reports of hybridisation in Australian stingless bees using multiple SNP markers. We find no evidence for contemporary or recent hybridisation, contrary to prior conclusions based on microsatellite data.

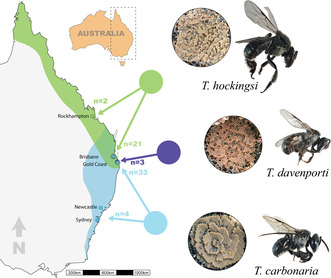

## 1 Introduction

The boundaries between species that diverged in allopatry can be tested when populations come into secondary contact through changes in habitat or climate, or as a result of anthropogenic movements across geographic barriers (Sánchez‐Guillén et al. 2013; Gloag et al. 2017; Brookes et al. 2020). The interpretation of hybridisation should be closely tied to the designation of species in population genetic terms (Paterson 1978). When species are considered conceptually as recombining gene pools (Ayala et al. 1994), hybridisation is the process of gene flow across two such species' gene pools. High levels of hybridisation in local areas of overlap between taxa are more congruent with their classification as subspecies (rather than species), which, by definition, have independent geographical distributions but hybridise freely where and when they do overlap (Walter 2003; Ford 1974). The frequency of hybridisation, or gene flow, is therefore key to understanding the limits of a given species gene pool, and to our understanding of speciation. The detection of true hybrids between two closely similar populations is especially sensitive to the molecular methodologies used, and more likely to be susceptible to sampling error because parent species are often similar in appearance to one another.

**FIGURE 1:**
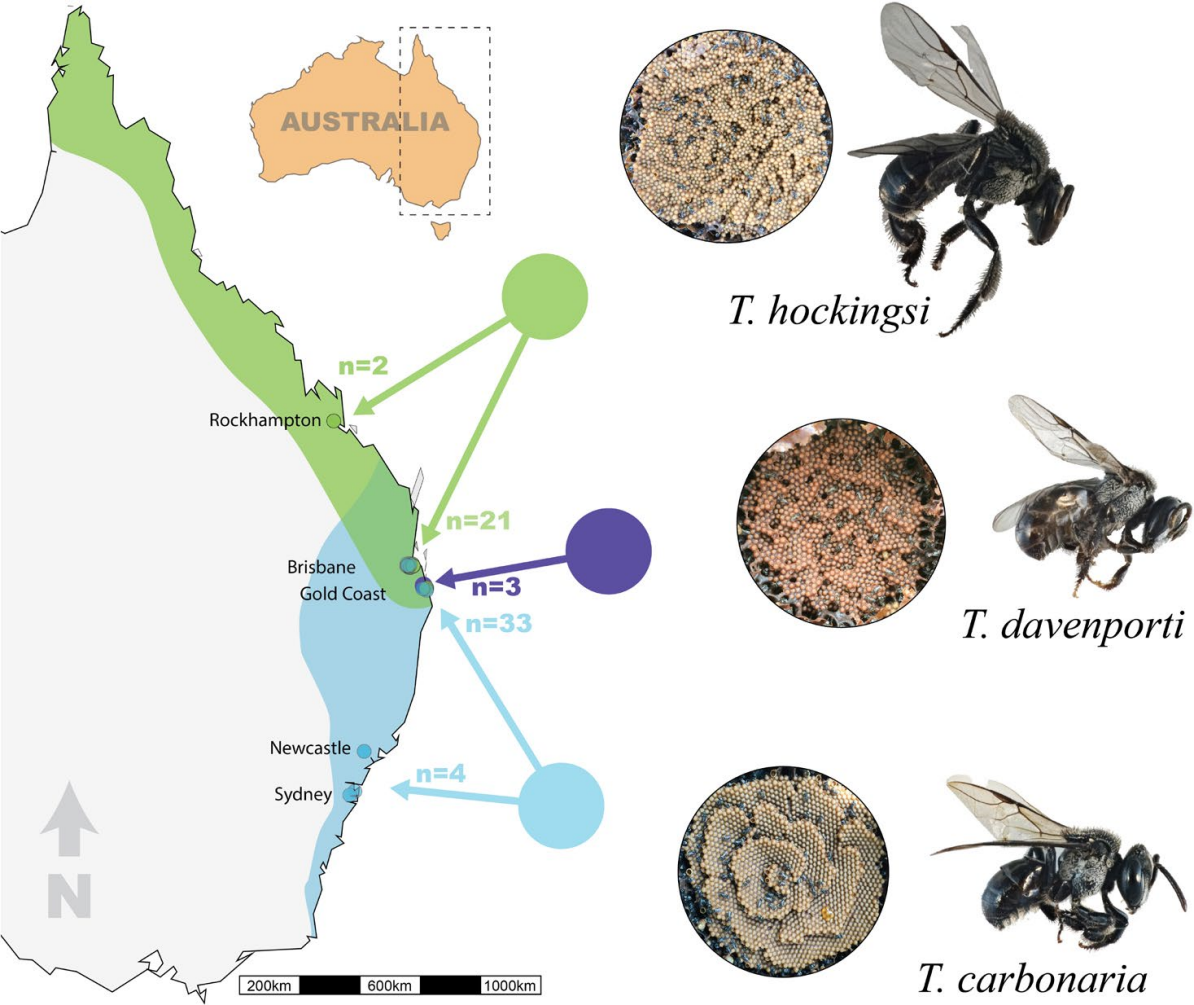
Map showing the known distribution of three species of *Tetragonula* in Australia. Sampling sites are shown as small, colored circles, and numbers of colonies used in this study are indicated. Images of the brood structure and the bees are shown on the right, and the distributions are taken from Heard (2016).

An Australian species complex of stingless bees (Meliponini) in the genus *Tetragonula* illustrates these challenges well. The genus *Tetragonula* includes three cryptic sister species (*T. carbonaria. T. hockingsi* and *T. davenporti* ), indigenous to eastern Australia, that are part of a monophyletic clade known as the ‘Carbonaria group’ (Franck et al. 2004). These bees can be kept readily in wooden hives, are often kept in suburban gardens, and are effective pollinators of several tropical and subtropical crops (Dyer, Streinzer, and Garcia 2016; Heard 1994). This has led to many thousands of colonies being kept by beekeepers in Eastern Australia. Today, the distributions of all three species overlap in parts of South East Queensland (Figure 1). Beekeeping and other human activities, such as land use change, have increased the overlap of their distributions in recent decades, particularly through the southward spread of *T. hockingsi* (Cunningham et al. 2014). Unfortunately, the historical distributions of the species are poorly known due to their closely similar morphology.

Each species cannot be reliably identified based on morphology alone, whether queens, workers (females) or drones (males) are considered (Dollin, Dollin, and Sagakami 1997). Rather, species are typically identified by the structure of their brood comb, with *T. carbonaria* producing a neat spiral of adjacent brood cells in a layered comb and *T. hockingsi* producing clusters of cells in a form known as semi‐comb (Figure 1). *Tetragonula davenporti* shares the semi‐comb brood structure of *T. hockingsi* and is known from only a handful of managed colonies in a sub‐coastal pocket of southern Queensland (Franck et al. 2004; Brito et al. 2014), but is believed to persist in the wild in nearby forested areas (P. Davenport pers. comm., 2019; R. Gloag unpublished data).

Relatively little is known about the mating behaviour of these bees because it occurs during the nuptial flight (Bueno, dos Santos, et al. 2022). Like all *Hymenoptera*, males are haploid while females (queens and workers) are diploid. Genetic data indicate that queens mate only once (Green and Oldroyd 2002), and observations of tethered virgins indicate that male genital detachment is likely the process by which this occurs (Smith 2020). Male mating congregations, which form outside colonies with virgin queens preparing for nuptial flights, can contain a mix of both *T. carbonaria* and *T. hockingsi* males (Cunningham et al. 2014). These two species are also known to engage in interspecific nest usurpations, in which an invading colony kills a resident queen and instals its own in the nest (Cunningham et al. 2014). This behaviour may increase the chances that males are attracted to queens of the other species. Males leave their natal colony and disperse through the environment searching for nests with virgin queens ready to mate (Bueno, Bueno, et al. 2022). They are thought to be attracted to requeening colonies based on the odour profiles such colonies emit (Bueno, dos Santos, et al. 2022). Following a nest usurpation, a mixed species colony can persist for some time (Lau et al. 2022), when males would presumably encounter mixed‐species odour signals at the colony. In this transition period, the structure of the brood comb may not accurately reflect the species of the resident bees. As stingless bees continuously dismantle and rebuild brood cells in a constant process, this transition period is likely to last around 50–90 days, (Heard 2016). In this paper we refer to a hybrid as a colony with one parent of one species and one parent of another, with all offspring having genetic material from both species (rather than a mixed species colony following a takeover).

Previous efforts to establish if gene flow occurs between these species, and at what rate, have returned equivocal results. Microsatellite markers have been used to look for genetic evidence of hybrids. Franck et al. (2004) identified some South East Queensland colonies of *Tetragonula* as being of hybrid origin (two of 130 total colonies sampled). This hybrid assignment was based on data from 13 microsatellite loci, where hybrids were defined as individuals that had a posterior probability of assignment of less than 90% to a cluster in an analysis using the genetic assignment software *STRUCTURE* (Pritchard, Stephens, and Donnelly 2000). The same three Australian *Tetragonula* species were re‐investigated by Brito et al. (2014), with nine microsatellite loci and two mitochondrial genes, and using a threshold of 95% posterior probability of assignment to a cluster in *STRUCTURE* to define hybrids. Three hybrid hives were reported within the zone in which *T. carbonaria* and *T. hockingsi* overlap in South East Queensland (from 235 total colonies sampled). They interpreted this hybridisation being a result of anthropogenic movement of northern *T. hockingsi* into southern Queensland. Brito et al. (2014) also included two hives reported to be *T. davenporti* in their study. One of the two *T*. *davenporti* hives included in their study clustered with *T. carbonaria* in a principal coordinate analysis (PCoA) of genetic distance based on the microsatellite data, and the other clustered slightly outside of the ‘ *T. carbonaria* ’ cluster. This led to the hypothesis that *T. davenporti* had hybridised with *T. carbonaria* at this site in the years between the two study of Franck et al. (2004) and Brito et al. (2014) and that one of the “*T. davenporti”* colonies was an F1 hybrid backcrossed to *T. carbonaria* .

Mitochondrial markers were also investigated in these previous studies, but these efforts were hampered by the amplification of nuclear copies of mitochondrial genes (numts) (e.g., for *cytochrome b* [*cytb*]; Franck et al. 2004). Recently, several numts of cytochrome oxidase subunit I (COI) were identified in *T. hockingsi* and *T. carbonaria* , which even led to the designation of *T. davenporti* as a true species being questioned (Françoso et al. 2019). Surprisingly, the genuine mitochondrial COI sequences of *T. hockingsi* and *T. carbonaria* are highly divergent (16.5%), relative to the divergence in the nuclear genes that have been sequenced (e.g., EF1alpha, Françoso et al. 2019). Subsequent analyses have revealed an unusual duplication of the entire mitochondrial genome (Françoso et al. 2023). This pattern of high mitochondrial divergence but low nuclear divergence might be consistent with relatively high levels of hybridisation leading to gene flow across the species and highlights the need for new analyses of nuclear gene flow to understand the species status of these bees and whether hybridisation occurs among them. This will in turn reveal the likely impact of continued movement of managed *Tetragonula* hives around Australia.

In this study, we aimed to resolve whether gene flow occurs between *T. carbonaria*, *T. hockingsi* and *T. davenporti* where they occur in sympatry in South East Queensland and to establish their species status. We took a multiple marker approach, using both microsatellite data (the same markers as previous studies; Franck et al. 2004, Brito et al. 2014), and a genotyping by sequencing approach that yielded 1745 SNP markers after filtering. We also look for evidence of mitochondrial introgression, by considering two nuclear gene sequences and one mitochondrial gene sequence of each species. We then use Illumina sequencing to reconstruct mitochondrial genomes and confirm that our mitochondrial sequences are not pseudogenes. We then examined regions of the nuclear genome flanking the Ef1 alpha and Opsin regions to further investigate the low variability. This approach reveals new insights about the extent of hybridisation in these bees, and their species status.

## 2 Materials and Methods

### 2.1 Sampling and DNA Extraction

We sampled four workers per colony from hived colonies of each species between February 2017 and April 2019 in South East Queensland ( *T. carbonaria* , *n* = 33 colonies, *T. hockingsi* , *n* = 21 colonies, *T. davenporti* , *n* = 3 colonies) and we also included two *Tetragonula hockingsi* hives from Rockhampton (Queensland), two hives of *T. carbonaria* from Newcastle (NSW), and two from Sydney (NSW); these latter two localities are outside the natural distribution of *T. hockingsi* and *T. davenporti* (Figure 1). Most *T. carbonaria* and *T. hockingsi* colonies were sampled in the greater Brisbane region. We opened each hive and assigned it to species based on brood nest architecture (spiral/layered comb or semi‐comb). The three *T. davenporti* colonies were sampled from the border area of Queensland and New South Wales and were the same colonies used for past genetic work on this species (P. Davenport, pers. comm., 2019). A further two *T. carbonaria* colonies and one *T. hockingsi* colony were sampled from the same meliponary as the three *T. davenporti* colonies (all three species are found at this one property). We extracted DNA using a silica spin‐column method (Ridley et al. 2016) and normalised concentrations to 5 ng/μL per sample following quantification (PicoGreen, Life Technologies, California, USA).

### 2.2 Nuclear Markers

*Microsatellites*: We used seven microsatellite loci (Green, Franck, and Oldroyd 2001) used in previous molecular studies of this group (Franck et al. 2004; Brito et al. 2014) to compare inference of hybridisation from both the microsatellites previously used and SNPS. We used PCR reactions of 2 μL DNA template, 0.5 U MyTaq polymerase (Bioline, Australia), 0.1 μM of forward primer, 0.2 μM of reverse primer, 0.2 μM M13 labelled primer with different fluorescent dyes (6‐FAM, VIC, PET or NED) and 1× buffer, with reaction conditions of an initial denaturation at 95°C for 3 min, followed by 10 cycles of 10 s at 95°C, annealing at 45°C for 25 s, and 1 min extension at 72°C, then 40 cycles of 10 s at 95°C, annealing at 55°C or 52°C for 25 s, and 1 min extension at 72°C, and the final extension was at 72°C for 2 min (see Cunningham et al. (2014) for locus‐specific annealing temperatures). Loci were then pooled with one locus per dye, cleaned with 1 U Exonuclease I and one unit of Antarctic Phosphatase, and genotyped by Macrogen Inc. (Seoul, Republic of Korea). Microsatellite peaks were analysed using the microsatellite plugin in Geneious Prime 2022.2.2 (http://www.geneious.com). We tested the loci for Linkage Disequilibrium using the probability tests implemented in Genepop version 4.7.5 (Rousset 2008) and found none of the pairwise tests returned significant results. Exact tests for Hardy Weinberg Equilibrium (HWE) were conducted for each locus in the Pegas package in R v 4.2 with 10,000 permutations (Guo and Thompson 1992; R core team 2022, Paradis 2010). All loci were in HWE.

*Single nucleotide polymorphisms*: We generated genome‐wide SNP data using a genotyping‐by‐sequencing method. We adapted a protocol and adaptor regime based on the methods of Elshire et al. (2011), Poland et al. (2012) and Peterson et al. (2012), with barcodes based on those of Caporaso et al. (2012). Samples were double‐digested with MspI and PstI. We then ligated 96 unique forward barcodes, and three different 96 well plates of reverse barcodes to the samples so that every sample had a unique forward and reverse inline barcode combination. Each plate of 96 samples was then pooled and size‐selected on a Blue Pippin (Sage Science, Beverly, USA) to 300–400 bp. Each of these pools was PCR amplified to complete the adaptors and add i7 indices. Full details of the method are provided in (Data S1; Table S1) and can be found online at http://www.jameshereward.org/GBS.html. We pooled 288 individuals per sequencing lane and sequenced the libraries with PE150 Illumina sequencing at Novogene (Beijing, China).

The sequence data were demultiplexed, assembled and SNPs called using STACKS v2.55 (Catchen et al. 2013). In the demultiplexing step we used the options ‐c ‐q ‐r to remove low quality sequences and correct barcodes. After optimising for the maximum number of loci in 80% of individuals we ran the *de novo* map pipeline with the optimised settings of ‐m 4 ‐M 3 and ‐n 3 (Paris, Stevens, and Catchen 2017). We removed loci with a heterozygosity higher than 0.65 and selected only one SNP per locus (the first) in STACKS. We then generated a VCF file and filtered it using vcftools. The data were filtered by setting a minimum minor allele count of three across the complete dataset (one heterozygote and one homozygote), which allows the conservative removal of singleton SNPs that are likely to be errors, without discarding too many legitimate rare alleles (Linck and Battey 2019). We set a minimum depth of five (mean coverage per locus was 78×) and kept only biallelic SNPs. We filtered the SNP markers for missing data in three steps. First, any marker with more than 50% missing data was discarded. Second, any individual that had data missing at more than 50% of the markers was discarded, to remove the individuals that had bad quality genotyping. Finally, any marker with more than 10% missing data was discarded to produce a final dataset with relatively little missing data and 1839 SNPs. To remove loci that were not in HWE we made two subsets of the VCF file, one containing a single individual per hive of *T. carbonaria* and one containing a single individual per hive of *T. hockingsi* (there were only three hives of *T. davenporti* ). We made a list of all markers that were out of HWE in each of these two species using a *p*‐value cut‐off of 0.05 in vcftools, which uses the exact test of Wigginton, Cutler, and Abecasis (2005). We then removed these markers from the main dataset leaving 1745 SNPs. We double checked that none of the remaining SNPs were in Linkage Disequilibrium by calculating the squared correlation coefficient with vcftools and using a threshold of *r* ^2^ = 0.5, none of the markers were reported to be linked. We then calculated the mean heterozygosity per individual across all SNPs using vcftools to confirm that all individuals are diploid and therefore female (the minimum heterozygosity was 0.47).

For both our microsatellite and SNP datasets, we calculated pairwise and overall *F*_ST_ using the Weir and Cockerham method implemented in the R package hierfstat and with 10,000 permutations (Weir and Cockerham 1984; Goudet 2004). We also performed principal component analyses (PCA) using the adegenet package in R (Jombart 2008) and *STRUCTURE* analyses (Pritchard, Stephens, and Donnelly 2000). *STRUCTURE* is an individual‐based clustering algorithm that assigns individuals to each of *K* population clusters using Hardy–Weinberg equilibrium and linkage information in genetic markers. For each *STRUCTURE* run, we used the admixture model and ran 500,000 iterations of burn‐in followed by 1 million iterations. We assumed three populations (*K* = 3) and performed 10 runs with different starting seeds using *STRUCTURE* threader (Pina‐Martins et al. 2017). These were then permuted and plotted using Clumpak server (Kopelman et al. 2015). We also used the Bayesian approach implemented in NewHybrids to test for the presence of specific hybrid categories (parental species, F1s, F2s and backcrosses) in both the microsatellite and SNP datasets using 100,000 iterations of burn‐in followed by 1 million iterations (Anderson and Thompson 2002). NewHybrids assumes two parental species, so we prepared three SNP datasets and three microsatellite datasets, each containing one pairwise species comparison (i.e., *T. carbonaria* vs. *T. hockingsi* , *T. carbonaria* vs. *T. davenporti* , *T. hockingsi* vs. *T. davenporti* ). For the SNP data we prepared these files by making three different population map files and using the populations module of stacks to read in the VCF and make three files in the *STRUCTURE* format, and then used PGD spider to convert these to NewHybrids format (Lischer and Excoffier 2012). The *STRUCTURE* and NewHybrids analyses were run for the SNP dataset with the HWE filter applied and the SNP dataset without the markers that were out of HWE removed, the results were the same for both datasets so only the HWE filtered analyses are presented.

### 2.3 Gene Sequencing

To confirm whether common marker genes showed species‐specific polymorphisms, we sequenced two nuclear genes (*EF1alpha* and *Opsin*) and one mitochondrial gene (*16S* rRNA). These genes were selected as they have previously been used for a phylogenetic study of stingless bees (Françoso et al. 2019; Rasmussen and Cameron 2009). For *EF1alpha* we used primers F2‐ForH (GGRCAYAGAGATTTCATCAAGAAC) and F2‐RevH2 (TTGCAAAGCTTCRKGATGCATTT) (Hines, Cameron, and Williams 2006), these primers amplify approximately 750 bp partially covering the gene and including 193 bp of intron and around 550 bp of coding sequence. For *Opsin* we used LWRhF (AATTGCTATTAYGARACNTGGGT) and LWRhR (ATATGGAGTCCANGCCATRAACCA) (Mardulyn and Cameron 1999), these primers yield around 680 bp covering part of the *Opsin* gene and this fragment includes two introns totalling 189 bp. For *16S* we used primers 874‐16S1R (TATAGATAGAAACCAAYCTG) (Cameron et al. 1992) and 16SWb (CACCTGTTTATCAAAAACAT) (Dowton and Austin 1994), which target 520 bp of the mitochondrial 16S rRNA (which has no introns). We performed PCRs in 25 μL reactions containing 2 μL DNA template, 1 U MyTaq polymerase (Bioline, Australia), 0.2 μM of each PCR primer, and 1x buffer. For amplification of *EF1alpha* and *16S* we performed PCR with 35 cycles at 52°C annealing, and for *Opsin* we used five cycles at 45°C annealing followed by 35 cycles at 50°C. PCR products were cleaned using 1 U of Exonuclease I and Antarctic Phosphatase (New England Biolabs, Ipswich, Mass., USA) and incubating at 37°C for 20 min followed by 10 min enzyme denaturation at 80°C. Sequencing was conducted using the same forward and reverse primers used for PCR, by Macrogen Inc. (Seoul, Republic of Korea). We edited sequence data with CodonCode aligner and aligned them using MAFFT in Geneious Prime 2022.2.2 (www.geneious.com). We generated haplotype networks using the TCS algorithm (Clement, Posada, and Crandall 2000) in PopART version 1.7 (http://popart.otago.ac.nz/) (Leigh and Bryant 2015).

### 2.4 Comparison of Mitogenomes and Nuclear Gene Regions Flanking EF1 Alpha and Opsin

The ‘Carbonaria group’ of *Tetragonula* are now known to have several pseudogenes (or ‘numts’, nuclear insertions of mitochondrial genes) (Françoso et al. 2019; see also Franck et al. 2004; Brito et al. 2014). To ensure we were amplifying only the true *16S* rRNA gene and to provide a reference genome for each species for future work, we conducted illumina genome sequencing on one individual of each of the three species and reconstructed the complete mitochondrial sequence. We also sequenced the genome and reconstructed the mitogenome for a *Tetragonula clypearis* sample collected in Cairns to use as an outgroup for the phylogenetic analyses. Sequencing libraries were made using the NebNext UltraII DNA kit for Illumina (New England Biolabs, Ipswich, Mass., USA), and PE150 sequencing conducted by Novogene (Beijing, China).

Sequences were checked with FastQC (Andrews 2019), then we removed adaptors, and quality‐trimmed the end of the sequences of all samples to q10 using bbduk from the bbtools package (version 36, Bushnell 2019). We used a kmer of 8 and reads with an average quality below 10 were discarded. We binned the sequences by depth using a kmer‐based approach in bbnorm (Bushnell 2019). We put everything under 500× coverage into one file (this includes the nuclear genome, which had between 23× and 51× coverage for each species, Table 1), everything between 500× and 3000× into another file (this is repetitive sequence and other artefacts below the depth of the mitochondria), everything over 3000× coverage was placed in a third file, and this contained mitochondrial sequence and anything else with a depth over 3000. This process excluded nuclear‐mitochondrial pseudogenes and reduced the amount of data going into subsequent steps. We then reconstructed mitogenomes by mapping the high depth reads to the complete (duplicated) mitogenomes of *T. carbonaria* and *T. hockingsi* (Françoso et al. 2023). For *T. davenporti* the rate of mapping to the *T. carbonaria* reference mitogenome was low, and the mitogenome was reconstructed using multiple rounds of mapping and extension to the longest contigs, and then joining of contigs and manual verification of the final sequence by read mapping (Geneious version 2022.2.2, https://www.geneious.com). This was also the case for *T. clypearis* .

**TABLE 1:** Characteristics of the genomes and their draft assemblies for three species of *Tetragonula* stingless bees, including genome size, coverage, and % repeat, which were estimated with a kmer analysis in bbnorm. The mitogenome coverage was assessed by read mapping, and the assembly statistics (length, longest contig, n50, number of contigs) were calculated in Quast and percent complete single copy orthologues calculated with BUSCO. The assemblies can be found at GenBank under the provided bioproject and biosample IDs.

Species	Genome size estimate (bp)	Nuclear genome coverage (X)	Mitogenome coverage (X)	% repeat	Assembly length (bp)	Longest contig (bp)	N50 (bp)	N contigs	Busco (C)	BioProject	BioSample
*Tetragonula carbonaria*	457,237,826	23	6899	33%	284,886,424	187,309	14,476	156,012	86.6%	PRJNA578948	SAMN13088838
*Tetragonula hockingsi*	376,762,141	35	11,089	26%	288,263,186	200,717	11,462	225,627	85%	PRJNA578994	SAMN13089661
*Tetragonula davenporti*	342,107,567	38	26,907	17%	288,842,417	271,029	18,924	72,464	87.8%	PRJNA578998	SAMN13089823

We aligned these four *Tetragonula* mitogenomes using MAFFT (Katoh and Standley 2013). We made a partition file for all the mitochondrial genes and used IQ‐TREE 1.6.12 to find the best partition scheme, select models, and perform maximum likelihood tree reconstruction using the MFP+MERGE option (Chernomor, von Haeseler, and Minh 2016; Kalyaanamoorthy et al. 2017; Minh et al. 2020). SH‐like approximate likelihood ratio test values (SH‐aLRT, Guindon et al. 2010) and ultrafast bootstrap approximation values (Hoang et al. 2018) were obtained in IQ‐TREE using 10,000 replicates each.

To investigate further the low nuclear divergence suggested by our two nuclear genes (*Opsin* and *EF1alpha*), we considered the remaining genome‐wide data generated from our Illumina sequencing. We took the lower genomic bin of the Illumina data (everything below 500× coverage based on kmer binning; Bushnell 2019) and assembled nuclear genomes for each species using SPADES (Bankevich et al. 2012). This was run with default settings, including the in‐built error correction module. We assessed the assemblies and generated basic statistics using QUAST (Gurevich et al. 2013) and BUSCO (Manni et al. 2021). We then took the 768 bp *EF1alpha* PCR product and searched it against custom‐blast databases that had been made from the genome assembly of each of the four species of stingless bee. We did the same for *Opsin*. These larger sequences (which contained the whole gene including introns and surrounding non‐coding sequence) were aligned with MAFFT, a partition file created, and a maximum likelihood tree was reconstructed with IQ‐TREE as described for the mitochondrial genomes in the previous section.

## 3 Results

### 3.1 Tests of Hybridisation Using Microsatellites and SNPs

**FIGURE 2:**
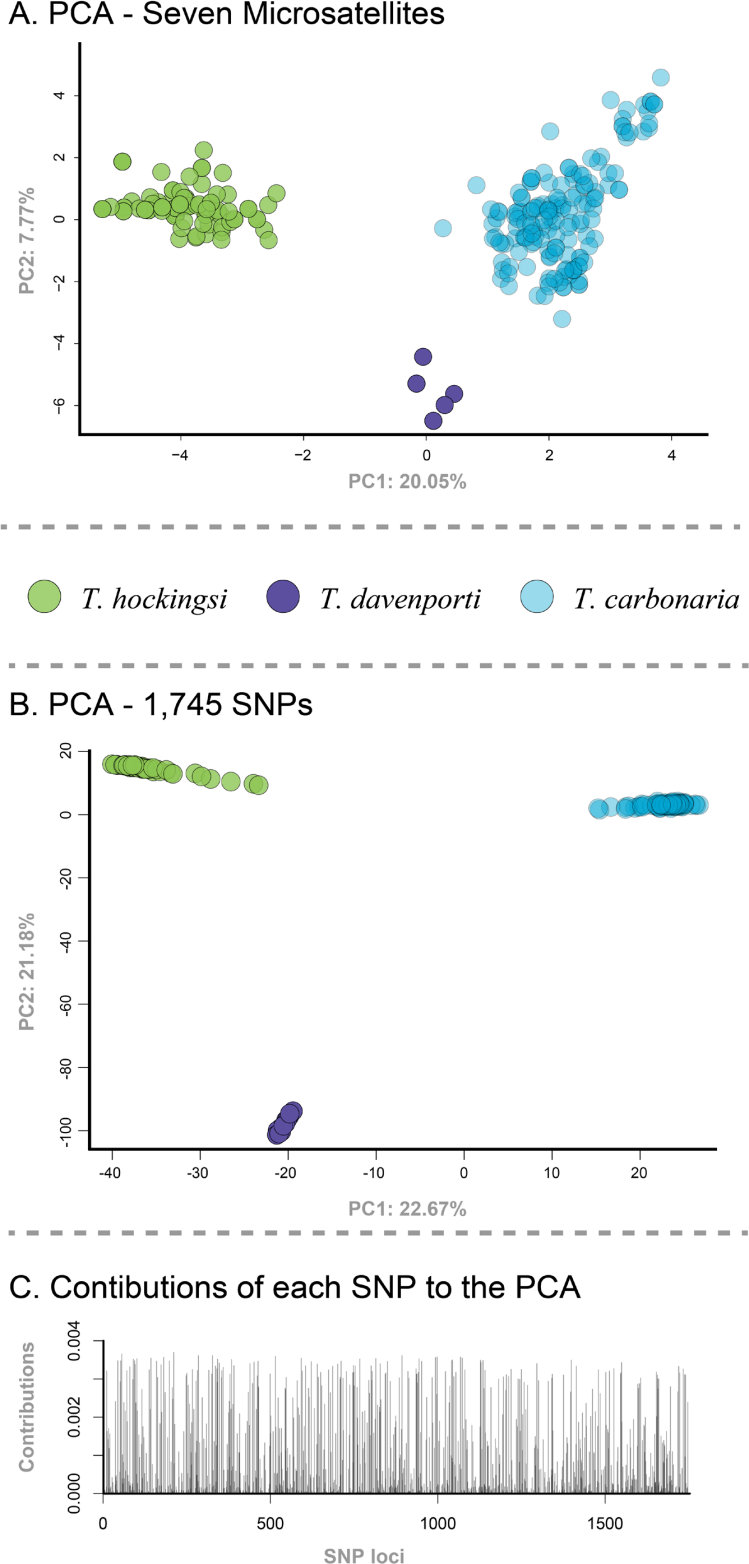
Principal Components Analysis (PCA) of genetic distance based on nuclear DNA markers. Plot showing principal components one and two based on the seven microsatellite markers (A), the same plot based on the 1745 nuclear SNP markers (B), and a loading plot showing the relative contributions of each of the 1745 SNP markers to the PCA (C).

Overall *F*_ST_ values were higher for the SNP dataset (0.675) than the microsatellite dataset (0.478), and the pairwise *F*_ST_ values between the three species were also higher for the SNP data (Table S2). PCAs using both the microsatellite and SNP datasets separated samples into three non‐overlapping clusters consistent with their species assignment (Figure 2). There was greater variation within each cluster in the microsatellite PCA (Figure 2A) compared to the SNP PCA (Figure 2B). The loading plot of the SNP data indicated that many of the 1745 SNP markers were contributing to the clustering in the PCA, rather than a few specific loci (Figure 2C). No individuals were placed between each of the parental species' clusters in either of the two plots (i.e., none was positioned where we might expect hybrids to be placed).

**FIGURE 3:**
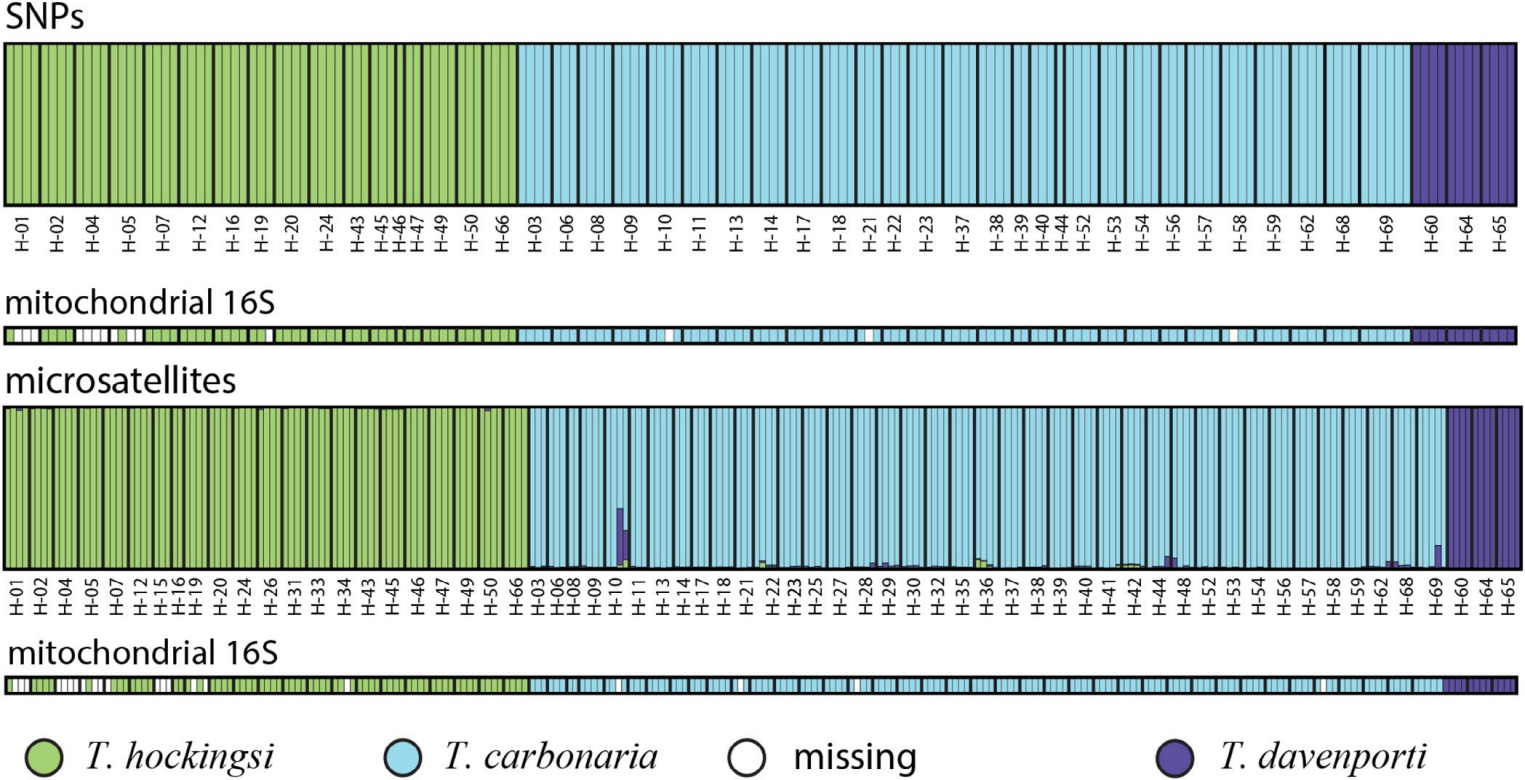
*STRUCTURE* plots assuming three hypothetical populations (*K* = 3), each bar represents an individual and the proportions of the three colors represent the posterior probability of that individual belonging to each of the three hypothetical (*K*) populations (species). The analysis was conducted based on 1745 SNPs (top) and for seven microsatellites (bottom), under each plot the species designation based on the mitochondrial 16S rRNA gene is shown.

**FIGURE 4:**
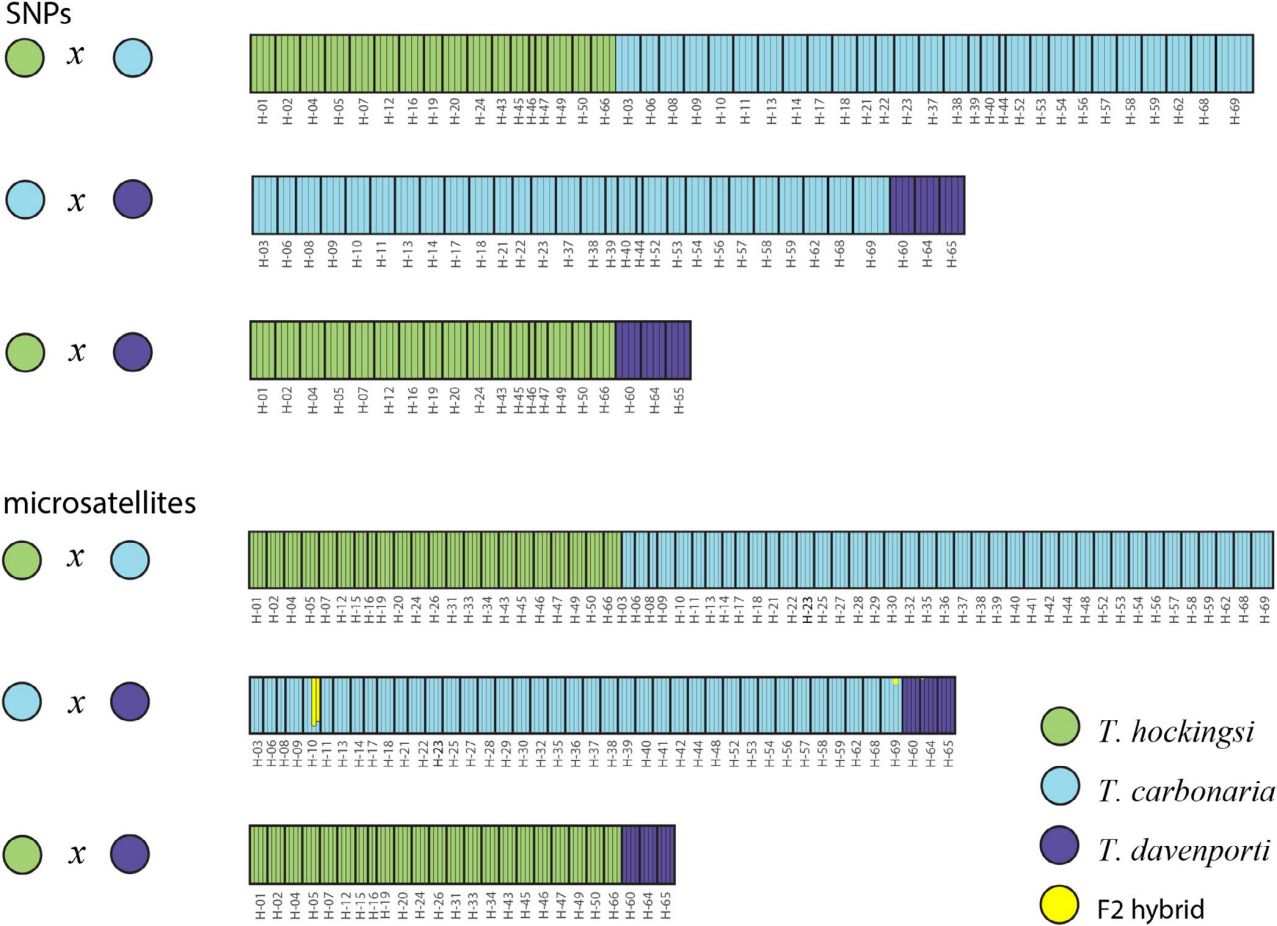
Plots of the NewHybrids analysis of the SNP data (top) and microsatellite data (bottom) for each of three pairwise species comparisons.

In the *STRUCTURE* analysis of the SNP data, all individuals were assigned to one of the three species with 100% posterior probability, and this corresponded to their assignment based on the mitochondrial *16S* rRNA sequence (Figure 3). The NewHybrids analysis also assigned all individuals to one of the two parental species included in each analysis with 100% posterior probability and no evidence of hybrids (Figure 4). For the microsatellite data, two *T. carbonaria* individuals showed a pattern of admixture in the *STRUCTURE* analysis (Figure 3) and were designated as F2 hybrids in the NewHybrids analysis (Figure 4). These same two individuals were present in the SNP dataset and were not inferred as hybrids based on those 1745 markers. Closer inspection of the genotypes of these two individuals revealed that they had specific alleles at one locus (Tc4.214) that were not present in other *T. carbonaria* individuals from our sample. When *STRUCTURE* was run on a version of the microsatellite dataset with this locus removed there was no longer a good separation of *T. carbonaria* and *T. davenporti* (Figure S1).

### 3.2 Species‐Specificity of PCR‐Amplified Gene Regions

**FIGURE 5:**
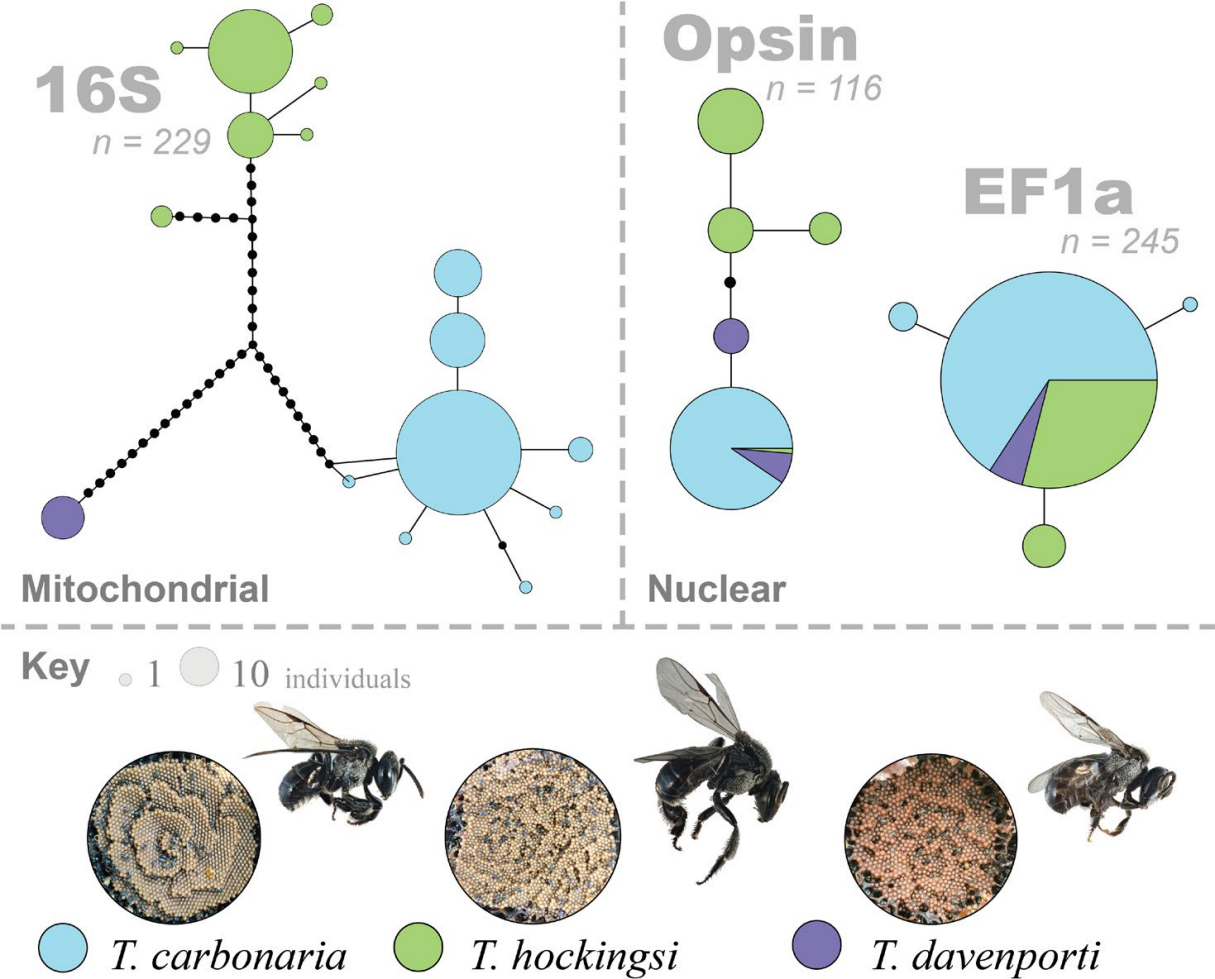
Haplotype networks for the mitochondrial *16S* rRNA gene, and nuclear *Opsin* and *EF1alpha* (top). Images of the brood and worker morphology of the three *Tetragonula* species are presented below.

Both the mitochondrial gene *16S* rRNA, and the nuclear gene *Opsin*, grouped samples into three haplotype clusters consistent with the species assignment based on nest structure (Figure 5), although pairwise divergence between species was greater for *16S*, with ~6% between *T. carbonaria* and *T. hockingsi* (based on 229 individuals representing 62 colonies) than for *Opsin*, with ~0.5% difference between *T. carbonaria* and *T. hockingsi* (116 individuals representing all 63 colonies). The interspecific sequence difference in the *16S* fragment was considerably lower than that of the complete mitogenome (see below) because half of the amplified region was highly conserved and had no variation. The nuclear gene *EF1alpha* had four haplotypes but identical sequence shared by all three species. No individuals showed mitochondrial‐nuclear discordance between *Opsin* and *16S*. That is, if an individual had a *16S* sequence representative of *T. carbonaria* it also had an *Opsin* sequence representative of *T. carbonaria* (Figure 5). However, one colony that was labeled *T. carbonaria* during field collections returned sequences and nuclear markers consistent with *T. hockingsi* and was assumed to be the result of either labelling error, or a recent interspecific colony takeover. Further, one colony suspected of being *T. davenporti* was genetically assigned to the *T. carbonaria* cluster, highlighting the difficulty of identifying these bees based on morphology.

### 3.3 Comparison of Mitogenomes and Nuclear Gene Regions Flanking EF1 Alpha and Opsin

**FIGURE 6:**
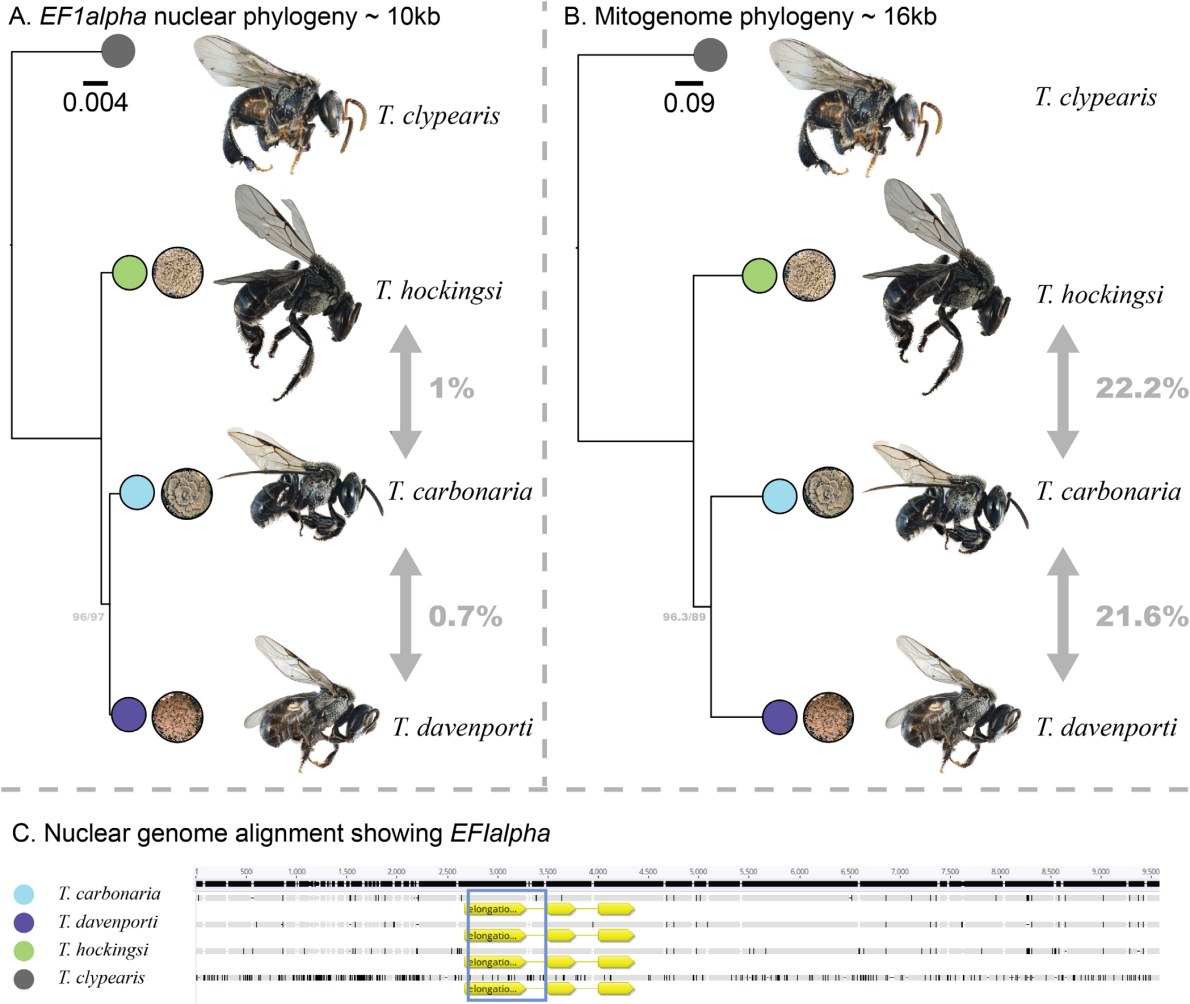
Maximum likelihood tree of four Australian *Tetragonula* species generated from 10 kb of DNA including the nuclear *EF1alpha* gene (A). Maximum likelihood tree constructed from whole mitogenomes (B). The nuclear genome alignment of *EF1alpha* and flanking regions. (C) Alignment of the four sequences, with the blue rectangle representing the PCR product and yellow arrows indicating coding sequence. Branch labels indicate the ultrafast bootstrap and SH‐aLRT values.

Our analyses of the mitogenomes for these three species indicated 22.2% pairwise differences across *T. carbonaria* and *T. hockingsi* , 21.6% across *T. carbonaria* and *T. davenporti*, and 23.6% across *T. davenporti* and *T. hockingsi* (Figure 6). Using these mitogenomes we were able to determine that the *16S* rRNA mitochondrial sequences were of mitochondrial origin and not nuclear pseudogenes.

Nuclear genomes returned a mean *n50* of 14,954 bp across the three species (Table 1). From this sequencing, we investigated further the lack of interspecific difference at *EF1alpha*. We took the *EF1alpha* sequence and used it as a ‘bait’ to pull out the regions of the genome surrounding the 768 bp PCR product. We managed to select at least 10 kb in each species that aligned around the PCR product using MAFFT (Katoh and Standley 2013). Only one Blast hit was found in each genome assembly (based on an *E*‐value threshold of 0.05) suggesting no duplications of these genes. Variation was detected around the *EF1alpha* locus, and this separated the three species, based on the one individual of each that we sequenced. We thus confirmed that the pairwise differences across these three bee species are indeed extremely low in this region of the nuclear genome (0.7%–1%, Figure 6) relative to that detected in the mitochondrial genome. Similarly, we recovered 26 kb around the *Opsin* gene, which returned the same low nucleotide difference between species (0.7%–1%).

Phylogenetic analysis based on either mitogenomes, or the flanking regions of *EF1alpha*, resolved the evolutionary relationships between the *Tetragonula* species in the same way, with *T. carbonaria* and *T. davenporti* diverging from each most recently, and *T. hockingsi* as their sister group (Figure 6).

## 4 Discussion

### 4.1 Testing for Hybridisation

Hybridisation and gene flow are central to our interpretation of species in population genetics terms, but accurately detecting hybridisation depends on the markers and analyses used. In the case of Australia's *Tetragonula* stingless bees, two previous studies concluded a low incidence of hybrid colonies in an area of sympatry between *T. carbonaria* , *T. hockingsi* , and *T. davenporti* (2%–3% of all sampled colonies; Franck et al. 2004, Brito et al. 2014). In this study, we use multiple additional molecular markers to analyse a new sample of colonies from the same area of sympatry (South East Queensland) and find all markers reliably segregate the three species, *T. carbonaria* , *T. hockingsi* and *T. davenporti* . Although we found no evidence of hybridisation in our data, we cannot discount rare occurrences of F1 hybrid colonies based on only 63 hive samples, but we find no evidence for gene flow across the species in this region and this indicates that if F1 hybrids do exist they do not produce further generations.

The lack of gene flow between *Tetragonula* species indicates that these species either do not mate, or that interspecific mating produces inviable or infertile offspring. Beekeepers regularly keep multiple species in close proximity, providing opportunities for interspecific mating. For example, at one property that we sampled, colonies of all three species had resided together for at least 9 years. In stingless bees, mating occurs on the wing close to the queen's colony. Males typically aggregate out the front of a colony that is undergoing the re‐queening process (i.e., has a virgin queen), presumably attracted by pheromones (Bueno, Bueno, et al. 2022; Vollet‐Neto et al. 2018). Male aggregations may attract dozens to hundreds of unrelated males from the surrounding area (Bueno, Bueno, et al. 2022; Bueno, dos Santos, et al. 2022; Cameron et al. 1992). Virgin queens then fly into the aggregation and mate, with just one male, before returning to the nest (Green and Oldroyd 2002). Even if aggregations sometimes contain males of more than one *Tetragonula* species, it may be that males are only sufficiently attracted to conspecific queens to attempt actual mating. Alternatively, queens may recognise the appropriate signals at the point a male makes contact with them, as in *Aphytis lingnanensis* parasitoids (Fernando and Walter 1997).

Interspecific mating may occur but produce inviable diploid brood due to genetic incompatibility. This latter scenario has been reported in sister species of honeybee that have come into sympatry recently ( *Apis cerana* and *Apis mellifera* ). Interspecific mating between these honeybees is common, but all hybrid offspring die at the early larval stage (Remnant et al. 2014; Gloag et al. 2017). In this case, mis‐mated queens may be quickly replaced. For example, workers of at least one stingless bee species ( *Scaptotrigona depilis* ) will execute laying queens that produce diploid male brood (Vollet‐Neto et al. 2017; Vollet‐Neto, Imperatriz‐Fonseca, and Ratnieks 2019). Clearly, further investigation into the mating of *Tetragonula* species is required to clarify the interactions between males and females in mixed species swarms.

Australian *Tetragonula* highlight several common reasons why it can be difficult to identify hybridisation between sister taxa. *Tetragonula* have nuclear pseudogenes of several common mitochondrial marker genes (Françoso et al. 2019; Franck et al. 2004). Such pseudogenes can complicate efforts to assess hybridisation through mito‐nuclear discordance, because they may be mistaken for valid haplotypes that are shared across species and thus mistakenly taken as evidence of introgression. Even when measures are taken to avoid pseudogenes, by checking for stop codons or frame‐shifting indels in their sequences, a subset of pseudogenes may still be missed. The reconstruction of whole mitogenomes from short read data, using a depth cut‐off to avoid nuclear copies, as in this study, may be necessary to identify true mitochondrial genes.

In *Tetragonula* stingless bees, pseudogenes have resulted in *T. hockingsi* and *T. carbonaria* appearing to have identical ‘mitochondrial’ sequences (Franck et al. 2004). In the study of Brito et al. (2014), apparent hybrids were classified as *T. carbonaria* based on their mitochondrial COI sequence, but these sequences were later revealed to be numts by Françoso et al. (2019). Excluding these pseudogenes provides confidence there is no mito‐nuclear discordance in our dataset and clarifies the true relationship between these three species.

A further issue is that the pattern expected from mito‐nuclear discordance (a sample returning one mitochondrial sequence and a different nuclear one) is the same as one gets when samples are mislabelled. Rates of mislabelling have been estimated at around 4% in published genetic datasets (Zych et al. 2017), so any cases of mito‐nuclear discordance at these rates probably deserve additional scrutiny. We detected one instance of mislabelling across two of our datasets, and we were able to locate a spreadsheet transposition error as the cause. Both pseudogenes and ‘mis‐labelling introgression’ have likely led to mistaken assignments of hybridisation in other taxa and may have inflated perceptions of the extent to which species hybridise.

Where nuclear markers suggest low divergence between species, the interpretation of *STRUCTURE* analyses must avoid biasing group assignment or over‐interpreting *STRUCTURE* plots (Lawson, van Dorp, and Falush 2018). *STRUCTURE* assignments using microsatellites were the basis of hybrid identification in previous studies of Australian *Tetragonula* and may have been sensitive to these issues, especially because of the relatively low number of markers used (Franck et al. 2004; Brito et al. 2014). In the study of Brito et al. (2014) the PCA plot indicated that there were no hybrids between *T. carbonaria* and *T. hockingsi* , in contrast to the conclusions drawn from the STRUCTURE analyses. In our study, the SNP data revealed no hybridisation, but the analyses based on the seven microsatellites did suggest that two individuals may have been hybrids. This signal in the microsatellite data was found to be driven by only one of the seven markers. When this marker was removed from the analysis it was no longer possible to fully distinguish *T. carbonaria* from *T. davenporti* in the *STRUCTURE* analysis (Figure S1). This highlights how inferences can be readily biassed when only a few markers are used, and the danger of over‐relying on STRUCTURE analyses as evidence for hybridisation. Simulation studies indicate that, depending on the *F*_ST_, 1–24 microsatellites are required to identify hybrids with *STRUCTURE* and NewHybrids (Vähä and Primmer 2005), yet many microsatellite‐based studies of hybridisation have used fewer than this required number. Indeed, in taxa where nuclear genome divergence is low, using many markers may be the only way to confidently assess hybridisation rates. Our results indicate that cases of hybridisation inferred from relatively few microsatellite markers should probably be reassessed with SNP data now that genotyping by sequencing assays are so readily available. The greater resolution provided by many SNP markers may lead to lower estimates of contemporary hybridisation in other systems where hybridisation has been inferred based on microsatellites previously.

### 4.2 Mitochondrial Divergence Between Tetragonula Species

Our mitogenome data support previous studies indicating a high mitochondrial sequence divergence between species of the ‘Carbonaria complex’ (Cunningham et al. 2014; Françoso et al. 2019, 2023). This mitochondrial divergence contrasts with the low divergence we observed in two nuclear genes and their flanking regions, around 0.7%–1% bp difference versus 21%–23% in the mitogenomes. We investigated the lack of variation in the *EF1alpha* PCR product to see whether it was restricted to this 700 bp fragment. We were able to recover the sequence flanking this region from our whole genome assemblies. These larger fragments did exhibit variation and recovered the same phylogeny as the mitogenomes. The discrepancy in divergence might indicate a particularly high mutation rate for mitochondria in this clade. The ratio of mitochondrial to nuclear mutation rate varies substantially across metazoans (from at least 0.8× to 40×, Allio et al. 2017). A mutation rate in *Tetragonula* mitochondria that was around 20 times that of their nuclear genome would be within the range reported for insects, albeit at the high end (Allio et al. 2017; Françoso et al. 2019). Several other Hymenoptera have high rates of mitochondrial divergence, compared to other insects (Castro, Austin, and Dowton 2002; Kaltenpoth et al. 2012). This pattern of mito‐nuclear substitution rate difference could alternatively be interpreted as being evidence for a relatively deep divergence time for these species (evidenced in the divergent mitochondria) that was later followed by a period of nuclear gene flow, erasing nuclear divergence. Under this scenario, past gene flow between diverged populations must have occurred in a similar fashion between all three species, because they share a common ratio of mitochondrial to nuclear divergence. Distinguishing between these two alternatives requires a better understanding of rate variation in mitochondrial and nuclear genomes of stingless bees relative to other *Hymenoptera*, and population genomic studies to elucidate the evolutionary history of this group.

*Tetragonula davenporti* remains the most mysterious of the three *Tetragonula* species considered in this study. It has been confirmed genetically from only one property in the Gold Coast hinterland (originally sourced from forested areas nearby and included in this study) and one wild specimen from the Queensland‐New South Wales border (R. Gloag, unpublished data). It is essentially identical in morphology with *T. carbonaria* and in nest structure with *T. hockingsi* . Nevertheless, the distinctive mitogenome of *T. davenporti* and lack of hybridisation reported here lends strong support to its status as a valid species. The natural range of *T. davenporti* is currently unknown, and the difficulty in identifying it morphologically to species means that its presence among managed colonies is also unknown. South East Queensland is an area of high endemicity, so it may well be endemic in this area.

This study investigated managed colonies mostly from Brisbane and SE Queensland because this is where hybridisation is most likely between these three species and has been reported previously. However, *T. carbonaria* and *T. hockingsi* also co‐occur in parts of Far North Queensland, and *T. hockingsi* is increasingly being transported by beekeepers into more southern parts of *T. carbonaria* 's range (pers. obs. TJS). Examining wild populations of this genus may reveal additional species within the ‘*carbonaria* complex’. Comparing wild populations to those that have been transported anthropogenically will provide further insight into the impact of human movement on the evolution of this group.

## Author Contributions

**James P. Hereward:** conceptualization (lead), data curation (lead), formal analysis (lead), investigation (lead), methodology (lead), visualization (lead), writing – original draft (lead), writing – review and editing (equal). **Tobias J. Smith:** conceptualization (supporting), investigation (supporting), methodology (supporting), writing – review and editing (supporting). **Ros Gloag:** conceptualization (supporting), formal analysis (supporting), investigation (supporting), methodology (supporting), visualization (supporting), writing – review and editing (supporting). **Dean R. Brookes:** conceptualization (supporting), methodology (supporting), visualization (supporting), writing – review and editing (supporting). **Gimme H. Walter:** conceptualization (supporting), supervision (supporting), writing – review and editing (supporting).

## Conflicts of Interest

The authors declare no conflicts of interest.

## Supporting information


**Figure S1.** *STRUCTURE* plot of the microsatellite data with locus 4.21 removed.

**Data S1.** Protocol for the genotyping by sequencing assay.

**Table S1.** Spreadsheet of the GBS adaptors.

**Table S2.** Pairwise *F*_ST_ values across the three species based on microsatellite and SNP data.

## Data Availability

The PCR amplicon data can be found on Genbank; acessions MN659448—MN659676 for 16S rRNA, MN659677—MN659921 for EF1alpha and MN659922—MN660037 for Opsin. Individuals used for whole genome sequencing have NCBI Biosample numbers as shown in Table 1. Illumina data has been deposited at the Sequence Read Archive (SRA), *T. carbonaria* = SRX7089578, *T. hockingsi* = SRX7104558, *T. davenporti* = SRX7104558. Genome assemblies can be found on Genbank, *T. carbonaria* = GCA_010645115.1, *T. hockingsi* = GCA_010645185.1, *T. davenporti* = GCA_010645165.1. These can all be found on the University of Queensland espace as well (Hereward et al. 2024, doi: https://doi.org/10.14264/84de1f8). Microsatellite data, raw SNP data, VCF files, R scripts, GBS analysis commands (stacks and vcftools), additional genome assembly data, mitochondrial genomes, and locations and dates of samples can be found on the University of Queensland espace (Hereward et al. 2024, doi: https://doi.org/10.48610/2ed1d54) The locations are only accurate to around 1 km to protect the privacy of beekeepers.
