# Supplementary figures and images for: Reassessing Hybridisation in Australian Tetragonula Stingless Bees Using Multiple Genetic Markers
Source: Ecol Evol. 2025 Jan 29;15(2):e70912. doi: 10.1002/ece3.70912 (PMC11775563; doi:10.1002/ece3.70912)

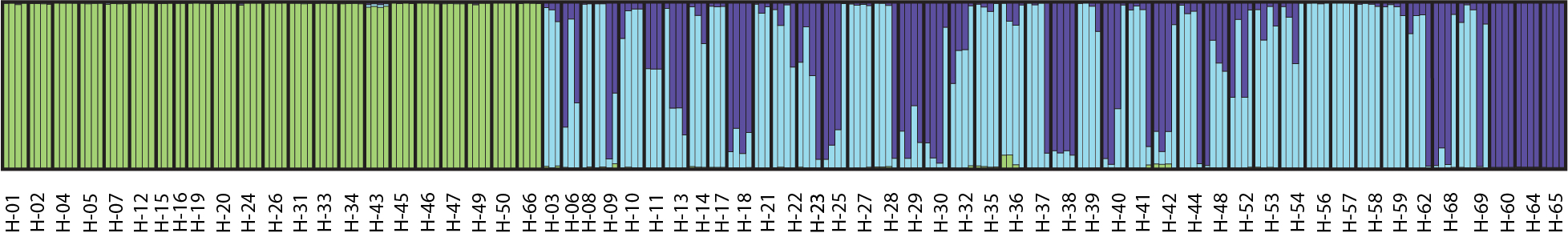

Supplement: Supplementary file 1 — Figure S1. STRUCTURE plot of the microsatellite data with locus 4.21 removed. [file ECE3-15-e70912-s004.jpg]
